# Potential Association between Subclinical Hypothyroidism and Childhood Migraine

**DOI:** 10.3390/medicina58101346

**Published:** 2022-09-25

**Authors:** Mohammed Abd Elmalik Hassan, Hussein Awad El-Gharieb, Mohamed Nasr, Wagih M. Abdelhay, Tahseen Samir Mohammed Yousef, Hossam M. Farid El-Zamek, Ahmed M. Zidan, Mohamed Nady, Mona A. Abdel-Kareem, Abdulkarim Hasan

**Affiliations:** 1 Pediatrics Department, Faculty of Medicine, Al-Azhar University, Cairo 11884, Egypt; 2 Neurology Department, Faculty of Medicine, Al-Azhar University, Cairo 11884, Egypt; 3 Histology Department, Faculty of Medicine, Al-Azhar University, Cairo 11884, Egypt; 4 Public Health and Community Medicine Department, Faculty of Medicine, Al-Azhar University, Cairo 11884, Egypt; 5 Clinical Pathology Department, Faculty of Medicine, Al-Azhar University, Cairo 11884, Egypt; 6 Radio-diagnosis Department, Faculty of Medicine, Al-Azhar University, Cairo 11884, Egypt; 7 Otorhinolaryngology Department, Faculty of Medicine, Al-Azhar University, Cairo 11884, Egypt; 8 Anatomy Department, Faculty of Medicine, Kafr Elsheikh University, Kafr Elsheikh 33516, Egypt; 9 Departments of Pathology, Faculty of Medicine, Al-Azhar University, Cairo 11884, Egypt

**Keywords:** migraine, hypothyroidism, subclinical hypothyroidism, goiter, pediatric

## Abstract

*Background and Objectives:* Migraine is caused by genetic susceptibility that is triggered by environmental as well as biological factors, and it is also linked to many somatic comorbidities, including clinical and subclinical hypothyroidism. We aimed to estimate the potential association between subclinical hypothyroidism (ScH) and migraine in children at our tertiary hospital. *Materials and Methods:* Using a case–control strategy, 200 children and adolescents were assigned to two equal groups: a case group (patients with migraine) of 100 patients and a control group of 100 patients without migraine. Clinical and biochemical parameters (TSH, FT4) were compared between the groups using statistical analysis. *Results:* Thyroid function comparison between the groups showed higher TSH but normal FT4 among children with migraine headache compared to the control group, which means more frequent ScH cases among the migraine group relative to the control (17% vs. 2%, *p* < 0.001). Obesity and overweight were more frequent among patients with migraine than the control group (8 and 5% vs. 2 and 1%, respectively). The (overweight/obese) patients with migraine had about 77% ScH and 15.4% overt hypothyroidism compared to 8% ScH and no overt hypothyroidism among normal body weight migraine patients (*p* < 0.001). No significant difference in the prevalence of nodular goiter between patients with migraine and controls was found. *Conclusions:* Based on our results, subclinical hypothyroidism is significantly linked to childhood migraine. Obesity and being overweight are more frequent among patients with migraine. Therefore, it may be logical to test the thyroid function in migraineur children, especially those with high BMI. Further studies are recommended to discover the mechanism of this association in children.

## 1. Introduction

Migraine is the most prevalent type of primary headache disorder among children: between 5% and 40% [1,2]. This rises with age, reaching 80% when patients are transferred to adult neurological consultation. Its attacks in childhood have a considerable consequence on the patient’s quality of life [1,2,3]. Migraine is caused by genetic susceptibility that is triggered by environmental as well as biological factors, and it is also linked to many somatic and psychic comorbidities [4].

On the other hand, subclinical hypothyroidism (ScH) is a laboratory finding where thyroid stimulating hormone (TSH) level is higher than the upper normal limit while free tetraiodothyronine (FT4) level is within the normal range [5,6]. In adolescents and children, the prevalence of ScH is <2% [7,8]. Pediatric ScH could be attributed to glandular and extraglandular etiologies, e.g., Down syndrome, Turner syndrome, cystic fibrosis, celiac disease, chronic renal insufficiency, antiepileptic drugs, and Hashimoto’s thyroiditis. Nevertheless, in many children, there is no obvious cause (idiopathic ScH) [9].

The clinical manifestations of ScH might range from no symptoms or signs to the full-blown picture of hypothyroidism. Being overweight is a clinical sign that is frequently linked to childhood ScH [1,9]. ScH may proceed to frank hypothyroidism or return to an euthyroid state over time. Nevertheless, in most children, it stays stable for a long time, especially in those with mild idiopathic ScH [10,11,12].

Surprisingly, few studies have found that migraine is linked to an increased incidence of both clinical and subclinical hypothyroidism [13,14,15,16,17]. L-thyroxin therapy results in a considerable reduction in headache frequency in patients with ScH [18]. Although the first article about this link was published in 1991, there has been ambiguity in the pathophysiological basis of this association until now [15,19]. Many theories related to mutual immunological, genetic, and environmental factors have been introduced, but all of them still lack strong support [13,14,15]. Other studies explain this comorbidity by the participation of thyroid hormones in pain-threshold control as well as TSH receptors that exist in the brain cortex and vessels [14,20].

The probable link between childhood migraine and ScH has gotten a lot of attention recently, but the evidence is inconclusive [21,22]. The aim of this work is to assess the incidence of ScH in 6- to 18-year-old children and adolescents with migraine.

## 2. Methods

### 2.1. Study Population

This case–control study was carried out in the neurology, otorhinolaryngology and pediatric outpatient clinics of Al-Azhar University Hospitals (El Hussein University Hospital and Sayed Galal University Hospital) in Cairo, Egypt between May 2021 and March 2022. The study included 200 children and adolescents assigned to two equal groups: a case group of 100 patients with migraine and a control group of 100 patients matched for sex and age without migraine.

The inclusion criteria comprised children and adolescents with migraine of both sexes aged 6–18 years. Patients without complete clinical data or refusing the study investigations were excluded. Neurological abnormalities, PedMIDAS disability score < 10 (see later), pregnancy, underweight, chronic debilitating diseases, and use of thyroid gland-affecting drugs, such as propranolol, constituted the exclusion criteria.

The diagnosis of migraine was based on the International Classification of Headache Disorders (ICHD)-III beta criteria, third edition [23].

The study was explained to all of the subjects or parents, and written agreement was obtained. This study was approved by the local ethics committee in accordance with the Declaration of Helsinki code of ethics for human studies.

Sample-size calculation was carried out by using Epi Info based on test power of 95%, confidence level 95%, ratio of exposed to unexposed 1, outcome in unexposed 40%, and outcome in exposed 65%, giving a minimum sample size of 200 participants: 100 exposed and 100 unexposed.

### 2.2. Clinical Evaluation

All patients underwent a general and neurological examination, as well as a comprehensive medical history that included headache character, age of onset, duration, frequency, aura, positive family history of migraine, and the use of variable migraine medications. The PedMIDAS questionnaire was answered by patients and their parents. It has been validated for migraine for those aged 4–18 years. The severity of migraine disability was stratified according to PedMIDAS score as follows: little to none, 0–10 (excluded from the study); mild, 11–30; moderate, 31–50; and severe, >50 [24].

### 2.3. Body Mass Index

Body mass index (BMI) is a simple and accurate method of assessing weight that can be used on all individuals aged 2–20 years [25]. BMI was calculated for each subject as weight (in kilograms) divided by height (in meters) squared (kg/m^2^) [26]. The readings were plotted on sex- and age-specific percentile charts and subject status classified accordingly as follows: obese with BMI ≥ 95th centile; overweight with BMI < 95th but ≥85th centile; nonobese with BMI < 85th but ≥5th centile; and underweight with BMI < 5th centile (excluded from the study) [27]. Sex- and age-specific Egyptian BMI percentile charts [26] were used in the study. The participants’ weight and height were measured while they were barefoot and dressed lightly. A digital electronic weighing scale as well as a wall-mounted stadiometer were used.

### 2.4. Thyroid Function Tests

All patients had a few milliliters of blood drawn in the morning, considering strict aseptic techniques. TSH and FT4 levels were determined using the Cobas 8000 e602 electrochemiluminescence assay.

TSH and FT4 had normal standard measures of 0.5–4.5 μIU/L and 0.7–2.00 ng/dl, respectively [28]. An elevated TSH (TSH > 4.5 μIU/L) while the FT4 is still within the normal range, has been characterized as ScH. Overt hypothyroidism was defined as decreased level of FT4 below the normal reference standard range.

### 2.5. Radiological and Histological Examination

During the clinical evaluation of the participants, we discovered that five patients had nodular goiter. Further assessment was done, and according to the guidelines, fine-needle aspiration biopsy (FNA) was performed [29].

### 2.6. Methodology of FNA

**Figure 1:**
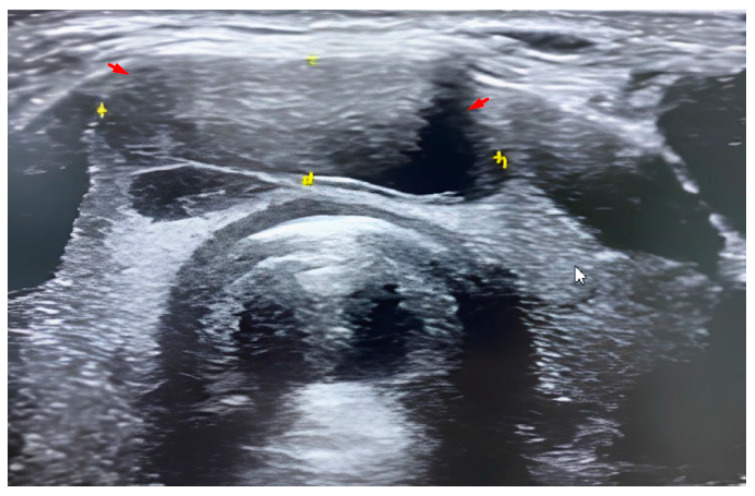
US imaging findings in benign lesion. Grayscale US image of the thyroid gland in a 16-year-old boy demonstrate isthmic solitary predominately cystic nodule with small solid component (red arrows).

The patient came into the procedure theater lying in a supine position with extended neck and antiseptically washed. In young children, numbing medicine and sedation was administered for two patients. An ultrasound transducer with a little bit of sterile water-soluble gel was applied to the patient’s neck. The needle was inserted through the skin under direct imaging guidance (Figure 1), advanced to the site of the thyroid nodule and aspirated samples of tissue. After the sampling, the needle was removed. Additional needles were reinserted only if additional samples were required by the cytologist, who rapidly examined the taken samples for adequacy.

Once the biopsy was complete, pressure was applied to the area to decrease the risk of bleeding with a bandage and the aspirated material was spread on multiple smears stained with rapid staining (Giemsa) and the rest of the smears were fixed in 95% alcohol, then stained with PAP to be ready for cytological examination by the cytopathologist or the histologist. The 2017 Bethesda System for Reporting Thyroid Cytology was followed.

One patient experienced a surgical removal (lobectomy) of a large thyroid nodule, which was sent for histopathology examination after excision.

### 2.7. Statistical Methods

Data are presented as means ± standard deviation (SD) for quantitative data or frequencies and percentages for qualitative data. Baseline and laboratory quantitative data comparisons were conducted between migraine cases and control using independent-sample *t*-tests after ensuring distribution normality using Kolmogorov–Smirnov and Shapiro–Wilk tests on SPSS software. Qualitative data were compared using either chi-squared or Fisher exact test as appropriate. Logistic regression analysis was done to determine the variables significantly associated with ScH in children with migraine. All tests were conducted at a 0.05 level of significance.

## 3. Results

Age distribution between the migraine and control groups did not show a significant difference (means 10.13 and 10.42 years, respectively). Sex distribution did not significantly differ between the groups either.

Obesity and overweight were more frequent among the migraineur group than the control group (8% and 5% vs. 2% and 1%, respectively). Thyroid function comparison between the groups showed higher TSH but normal FT4 among children with migraine headache compared to the control group, meaning more frequent ScH cases among the migraine group relative to the control (17% vs. 2%, *p* < 0.001). These data are shown in Table 1.

Further analysis of the migraine group according to the BMI and thyroid function tests showed that the overweight/obese patients with migraine had about 77% ScH and 15.4% overt hypothyroidism compared to 8% ScH and no overt hypothyroidism among normal-body-weight migraine patients (*p* < 0.001, Table 2).

The statistical analysis of the migraine cases (without overt hypothyroidism) according to thyroid function tests and migraine criteria showed an association between thyroid function and migraine disability, where 53% of migraine cases with ScH had moderate disability and 29.4% had severe disability compared to 29.6% moderate disability and 11.1% severe disability among migraine cases with normal thyroid function (*p* = 0.004). On the other hand, there was no association between thyroid function and occurrence of aura (*p* = 0.758) (Table 3).

Twenty-six patients showed migraine with aura; however, the rest (74/100) had no aura preceding migraine (Table 4)

Logistic regression analysis demonstrated that increased BMI was the only variable significantly associated with ScH, with *p*-value 0.001, odds ratio 1.020, and 95% CI 1.009–1.31, as shown in Table 5.

**Figure 2:**
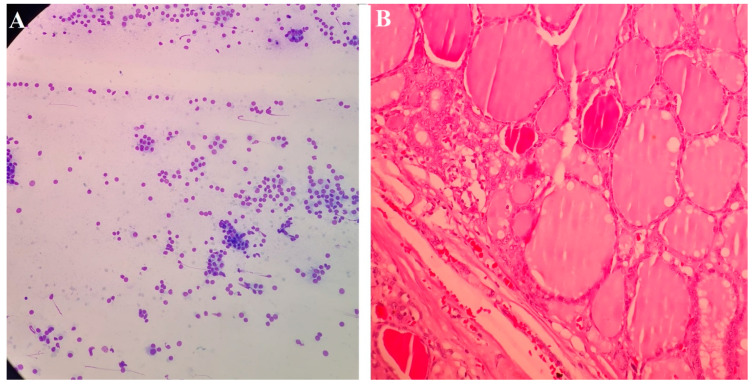
(**A**) Cytology of a case of nodular goiter showing bland-looking follicular cells (Giemsa stain). (**B**) Histopathology showing multiple follicles lined with cubical cells with round nuclei and filled with colloid with outer capsules and occasional hemorrhage (H&E, 100×).

During the medical evaluation of the participants, we noticed five patients had goiters. Further evaluation was performed, and according to the guidelines, all of them underwent FNA (four had a colloid nodule (diagnostic category II) and one had cellular atypia (diagnostic category III)) and underwent lobectomy in the general surgery department, then were examined at the pathology department with H&E-stained sections revealing thyroid nodular goiter (Figure 2).

## 4. Discussion

Our results showed a significantly higher prevalence of ScH among migraineur children and adolescents when compared to non-migraineur control participants (17% vs. 2%). The Fallah et al. study [21] is one of the few to look into the link between ScH and migraine, which was undertaken in 5- to 15-year-old children and noted a higher prevalence rate than ours (24%). That study discovered a 24% prevalence of ScH among their migraineur patients, which was not supported by other surveys or research articles. Following that, the authors presented a study on the effectiveness of hormonal replacement therapy in those patients, finding that the L-thyroxin was beneficial in lowering migraine attacks [18].

Ekici and Cebeci noticed that ScH and migraine were not typically linked in adolescents and children, which contradicts our findings [22].

Conflicting data about the comorbidity of ScH and migraine in adult patients also exist. A recent Egyptian adulthood case–control study by Emad et al. noted a significantly higher prevalence of ScH among migraine adult participants than the control subjects (22.3% vs. 9.2%, *p* = 0.002) [13]. In another Egyptian adulthood study of 212 tension and migraine headache participants, Abou Elmaaty et al. discovered that the prevalence of ScH was 23.3% in the headache group and 9% in the control group [14]. According to Khan et al., 22% of the primary headache group participants had ScH and 7.2% had overt hypothyroidism compared to 11.2% of participants with ScH and 1.2% of participants with overt hypothyroidism in the control group [30].

Additionally, previous articles have found a greater risk of migraine in adults with ScH. In a study of 75 individuals with ScH, Rainero et al. discovered that 62% of them had migraines [31]. According to Lima Carvalho et al., 35% of ScH patients developed migraines [16].

On the other hand, only 1.3% and 0.4% of patients with migraine, respectively, had ScH and overt hypothyroidism, according to Turkish authors [32]. Another Norwegian studt found that increasing TSH levels reduced the frequency of headaches [33].

There are a variety of possible explanations for the link between migraine and ScH, including incompletely understood complex genetic, metabolic, immunological, and environmental interactions [17]. Mutual genetic and metabolic connections between migraine and ScH could exist. For example, in both migraine and ScH, higher serum levels of homocysteine and methyltetrahydrofolate reductase gene mutations may be found [17,34,35]. Genetic variations in genes modulating the immune response, such as the cytokines regulating genes and human leukocyte antigen genes, play a role in thyroid autoimmune disorders. Surprisingly, specific human leukocyte antigen genetic variants have also been discovered in patients with migraine [36,37].

In the pathogenesis of migraine, research has discovered considerable changes in the levels of regulatory T cells (CD4+ and CD25+) [38]. Excitingly, in experimental research, (CD4+ and CD25+) T cells have been found to be involved in the etiology of autoimmune thyroiditis [15].

Migraine-induced inflammation may increase the risk of autoimmune thyroiditis. In patients with migraine, some clinical clues show an increase in CRP and alterations in T lymphocyte proportions between episodes, as well as a significant increase in leukocyte adhesion and levels of cytokines [39]. In autoimmune thyroiditis, similar immunological alterations have been documented [17]. One of the primary causes of ScH, as reported by Taylor et al., is autoimmune thyroiditis [40]. We did not detect any patient diagnosed with autoimmune disease in this study that may have been due to their young age.

A prior research article noted that pollutants like bisphenol A can alter the thyroid peroxidase enzyme, which is important in thyroid hormone synthesis. Bisphenol A is one of the migraine’s triggers, according to Vermeer et al. [41,42].

The evaluation of migraine now must include a disability and quality-of-life assessment [24,43]. When patients with migraine with ScH were compared to patients with migraine without ScH, we found a substantial difference in migraine disability, showing a considerable ScH-related disability in patients with migraine. Comparable results were noted by Emad et al. [13]. ScH has been shown in earlier research to have an adverse influence on quality of life [44]. Fatigue, body aches, and mood and memory changes are potential causes of disability in ScH [45]. On the other hand, negative emotions, nervousness, and headache pain are potential causes of disability in migraine [46].

Obesity appears to have a direct relationship with migraine, with the latter either worsening headache attacks in patients with migraine or raising the likelihood of developing migraine [47]. At the same time, obesity has been linked to ScH, and obese patients may have mild hyperthyrotropinemia [48,49].

We found that patients with migraine had a greater BMI than the control group, so obesity and being overweight were significantly more common in the migraine group than in the control group. When patients with migraine with ScH were compared to patients with migraine without ScH, we found a substantially higher frequency of overweight and obese patients when both comorbidities existed together.

Interestingly, thyroid peroxidase antibody and thyroglobulin antibody have been evaluated in all cases with ScH and overt hypothyroidism, and both were negative. This is consistent with the findings of Bona et al. [50], who conducted a systematic review of eight articles to investigate the natural history of ScH in children, and concluded that ScH is a benign condition that does not affect anthropometric measurements or physiological puberty staging, and in most cases is a remitting process with a low risk of developing overt hypothyroidism, more frequently evolving toward euthyroidism, or continually remaining in a condition of isolated hyperthyrotropinemia. They also reported that the rate of development of overt hypothyroidism ranged between 0 and 12.5% in three studies.

On the other hand, obesity and being overweight can explain ScH in 10 of our cases with migraine, where studies indicate that ScH seems to be a result rather than a cause of weight gain [51]. Another explanation is iron-deficiency anemia, which is one of the causes of ScH, and its prevalence among our patients was about 45% in both cases and controls without significant difference.

There was no significant difference in the prevalence of goiter between patients with migraine and controls. Four patients with migraine showed thyroid nodules on clinical examination, and they were all advised for FNA according to the 2015 American Thyroid Association guidelines, all of them revealing benign features leading to follow-up, except one who underwent lobectomy according to the surgeon’s decision as thyroid nodules in children and adolescents are less common and need careful investigations and management, regardless of whether the child is symptomatic or asymptomatic [52]. Study limitations include lack of multicenter study assessment and inability to evaluate other related factors, such as levels of regulatory T cells.

## 5. Conclusions

Based on our results, subclinical hypothyroidism is significantly linked to childhood migraine. Obesity and being overweight are more frequent among patients with migraine. Therefore, it may be logical to test thyroid function in migraineur children, especially those with high BMI. Further studies are recommended to discover the mechanism of this association in children.

## Figures and Tables

**Table 1:** Characteristics and clinical data comparison between cases and controls.

	Migraine Cases	Control	*p*-Value
	*N* = 100 (%)	*N* = 100 (%)	
Characteristics:			
Age—mean ± SD (years)	10.13 ± 2.37	10.42 ± 3.51	0.49
Male—number (%)	54 (54)	52 (52)	0.78
Female—number (%)	46 (46)	48 (48)	
Family history of thyroid disorders			
Negative	97 (97)	99 (99)	0.621
Positive	3 (3)	1 (1)	
BMI—mean ± SD	25.5 ± 4.84	24.31 ± 3.31	0.044 *
BMI categories—number (%):			0.03*
Normal	87 (87)	97 (97)	
Overweight	8 (8)	2 (2)	
Obese	5 (5)	1 (1)	
Laboratory values—mean ± SD:			
FT4 (ng/dl)	1.61 ± 0.23	1.65 ± 0.30	0.291
TSH (μIU/L)	3.94 ± 1.63	3.18 ± 0.99	<0.001 *
Thyroid function—number (%):			<0.001 *
Normal	81 (81)	98 (98)	
Subclinical hypothyroidism	17 (17)	2 (2)	
Overt Hypothyroidism	2 (2)	0 (0)	
Goiter			0.175
No goiter	96	99	
Goiter	4	1	

BMI: body mass index, FT4: free thyroxine, TSH: thyroid-stimulating hormone. Data are expressed as either means ± SD for quantitative data or frequencies and percentages for qualitative data. Statistical analyses were conducted using *t*-tests for quantitative data and chi-squared/Fisher exact test for qualitative data.* Significant at *p*-value of 0.05.

**Table 2:** Subgroup analysis of migraine cases according to body mass index.

	Normal Body Weight	Overweight/Obese	*p*-Value
	*N* = 87 (%)	*N* = 13 (%)	
Thyroid function—number (%):			
Normal	80 (91.95)	1 (7.69)	<0.001 *
Subclinical hypothyroidism	7 (8.05)	10 (76.92)	
Overt Hypothyroidism	0 (0)	2 (15.38)	

Data are expressed as frequencies and percentages. Statistical analysis was conducted using Fisher exact test.* Significant at *p*-value of 0.05.

**Table 3:** Subgroup analysis of migraine cases according to migraine subtypes and severity.

Migraine Cases without Overt Hypothyroidism (n = 98)			
	ScH	Normal Thyroid	*p*-Value
	*N* = 17 (%)	*N* = 81 (%)	
Migraine subtypes			
With aura	4 (23.5)	22 (27.2)	0.758
Without aura	13 (76.5)	59 (72.8)	
PedMIDAS score			
Mild	3 (17.6)	48 (59.3)	
Moderate	9 (52.9)	24 (29.6)	0.004 *
Severe	5 (29.4)	9 (11.1)	
Age at onset—mean ± SD (years)	8.93 ± 1.34	9.12 ± 1.20	0.562
Duration of migraine—mean ± SD (months)	13.83 ± 2.58	13.53 ± 2.61	0.667

Data are expressed as frequencies and percentages. Statistical analysis was conducted using Fisher exact test. * Significant at *p*-value at 0.05.

**Table 4:** Subgroup analysis of thyroid function, comparing migraine with aura and migraine without aura vs. controls.

	Normal Thyroid	ScH	Overt Hypothyroidism	*p*-Value
Migraine with aura (N = 26)	22	4	0	0.016 *
Control (N = 100)	98	2	0	
Migraine without aura (N = 74)	59	13	2	<0.001 *
Control (N = 100)	98	2	0	

Statistical analysis was conducted using Fisher exact test.* Significant *p*-value at 0.05.

**Table 5:** Logistic regression analysis for variables associated with ScH in migraineur children.

	B	SE	Wald	*p*-Value	Odds Ratio	95% CI for Odds Ratio	
						Lower	Upper
BMI	0.017	0.004	11.532	0.001	1.020	1.009	1.031
Age of onset (years)	−0.005	0.016	0.110	0.742	0.996	0.962	1.032
Duration of illness (months)	−0.036	0.119	0.079	0.765	0.941	0.765	1.209
Sex	0.479	0.577	0.719	0.344	1.609	0.536	4.955
Positive family history of thyroid illness	0.772	0.570	1.809	0.188	2.209	0.704	6.971
Aura	0.275	0.165	2.665	0.980	1.320	0.938	1.875
